# Genomic Profiling of Blood-Derived Circulating Tumor DNA from Patients with Advanced Biliary Tract Cancer

**DOI:** 10.3389/pore.2021.1609879

**Published:** 2021-10-15

**Authors:** Chen Chen, Tao Wang, Mengmei Yang, Jia Song, Mengli Huang, Yuezong Bai, Hao Su

**Affiliations:** ^1^ Hepatobiliary Surgery, Hunan Provincial People’s Hospital (The First Affiliate Hospital of Hunan Normal University), Changsha, China; ^2^ The Medical Department, 3D Medicines Inc., Shanghai, China; ^3^ Department of Hepatobiliary Surgery, First Affiliated Hospital of Guangxi Medical University, Nanning, China

**Keywords:** next-generation sequencing, signaling pathway, circulating tumor DNA, biliary tract cancer, genomic feature

## Abstract

**Background:** Biliary tract cancer is a highly lethal malignancy with poor clinical outcome. Accumulating evidence indicates targeted therapeutics may provide new hope for improving treatment response in BTC, hence better understanding the genomic profile is particularly important. Since tumor tissue may not be available for some patients, a complementary method is urgently needed. Circulating tumor DNA (ctDNA) provides a noninvasive means for detecting genomic alterations, and has been regarded as a promising tool to guide clinical therapies.

**Methods:** Next-generation sequencing of 150 cancer-related genes was used to detect gene alterations in blood-derived ctDNA from 154 Chinese patients with BTC. Genomic alterations were analyzed and compared with an internal tissue genomic database and TCGA database.

**Results:** 94.8% patients had at least one change detected in their ctDNA. The median maximum somatic allele frequency was 6.47% (ranging 0.1–34.8%). *TP53* and *KRAS* were the most often mutated genes. The frequencies of single nucleotide variation in commonly mutated genes in ctDNA were similar to those detected in tissue samples, *TP53* (35.1 vs. 40.4%) and *KRAS* (20.1 vs. 22.6%). Pathway analysis revealed that mutated genes were mapped to several key pathways including PI3K-Akt, p53, ErbB and Ras signaling pathway. In addition, patients harboring *LRP1B*, *TP53*, and ErbB family mutations presented significantly higher tumor mutation burden.

**Conclusions:** These findings demonstrated that ctDNA testing by NGS was feasible in revealing genomic changes and could be a viable alternative to tissue biopsy in patients with metastatic BTC.

## Background

Biliary tract cancer (BTC) is a heterogeneous group of malignancies including intrahepatic cholangiocarcinoma (IHC), extrahepatic cholangiocarcinoma (EHC) and gallbladder cancer (GBC), which account for 3% of gastrointestinal malignancies [1,2]. Despite being rare in western countries, the incidence of BTC is increasing worldwide [3,4]. BTC is an aggressive disease with a dismal prognosis [5]. Complete surgical resection provides the only chance for cure, but only 10% of patients are diagnosed at early-stage disease and are suitable for resection [6]. In addition, the recurrence rate is relatively high [7,8]. Thus, for the majority of BTC patients, systemic chemotherapy is the mainstay of treatment. Gemcitabine plus cisplatin (GemCis) is the standard regimen for first-line treatment, but the objective response rate is about 20% and the survival gain is limited [9]. These highlight the need for the development of more effective treatment strategies.

Several molecular profiling studies have characterized the genomic landscape of BTC and indicated potentially targetable genomic alterations, including *IDH1* mutations, *FGFR2* fusions, *BRAF* mutations and so on [10,11]. Based on results of large clinical trials, targeted therapy drugs pemigatinib and ivosidenib have been approved by FDA to treat cholangiocarcinoma patients with *FGFR2* fusions and *IDH1* mutations, respectively [12,13]. These demonstrated the necessity that all patients underwent genetic testing prior to initiation of treatment.

While tissue biopsy remains the gold-standard, tissue may not be available or limited for some patients. And the inter and intratumor heterogeneity is another pivotal challenge [14,15]. Liquid biopsy has emerged as a strategy to these challenges by detecting circulating tumor DNA (ctDNA) [16]. It is becoming a widely used diagnostic tool for identifying genomic alterations to guide therapy and prognosis. In BTC, several researches have been launched on assessing the sensitivity and positive predict value of ctDNA. Kinugasa et al. [17] revealed that there was high concordance rate between bile ctDNA and tissue DNA samples and ctDNA might be used as a tool to diagnose gallbladder cancer. In addition, changes in cell-free DNA correlated well with tumor marker dynamics in pancreatobiliary carcinoma, thus demonstrating the feasibility of cfDNA sequencing in identifying tumor-derived mutations [22].

In this study, we identified genomic alterations in blood-derived ctDNA from patients with BTC and assessed the concordance between alterations from ctDNA and tumor tissue DNA. Our aim is to prove that blood-derived ctDNA sequencing could be a potential complement to tissue testing, and might guide personalized cancer treatment.

## Materials and Methods

### Sample Collection and Clinicopathologic Data

From January 2017 to December 2018, blood samples from 154 patients and tumor specimens from 545 patients with metastatic BTC were collected for tumor genomic DNA (gDNA) sequencing in Hunan Provincial People’s Hospital. The parallel blood samples of those 545 tumor tissues were also collected to identify normal genomic DNA sequences. Hunan Provincial People’s Hospital Medical ethics committee approved this study (2019 Scientific Research Ethics Review NO: 71), and all patients signed the waiver of informed consent form. All these samples were sent to a commercial company owning a CLIA-accredited/CAP-certified laboratory (3D Medicines Inc., Shanghai, China) for gene panel sequencing. In addition, the clinicopathologic characteristics, age and sex, were collected.

### DNA Isolation and Sequencing

The methods of DNA extraction, sequencing and data analysis obeyed the published descriptions with some modifications [37]. Briefly, venous blood in STRECK tubes was centrifuged and kept the upper layer for the following tumor gDNA extraction via using the QIAamp Circulating Nucleic Acid Kit (Qiagen, Germany). The cfDNA libraries were established by Accel-NGS 2S Plus DNA Library Kit (Swift BioSciences, United States), and then sequenced. The gDNA of tissue sample with quality control and white blood cells were extracted by the DNeasy Tissue or Blood Kit (Qiagen, Germany), respectively. After fragmenting gDNA, the sequencing libraries were prepared by KAPA Hyper Prep Kit (KAPA Biosystems, United States). After capturing, the libraries were loaded into NextSeq500 platform (Illumina, United States) and performed next-generation sequencing (targeted) 150 cancer-related genes [18]. After eliminating duplicate or redundant information, the average coverage depth was 3000× for ctDNA and 500× for tissue sample.

### Data and Statistical Analysis

Sequencing reads were mapped to the GRCh37/hg19 human reference genome, and analyzed for somatic genomic alterations (GAs) including single nucleotide variant (SNV), copy number variation (CNV) and fusion. The range of maximum somatic allele frequency (MSAF) was defined among 0.1 and 35% for all the somatic alterations per sample. Variants of unknown significance was included for calculating MASF, however nor was single nucleotide polymorphism. Clinically relevant GAs were defined as GAs that associated with response to currently available therapies or in target-driven clinical trials. TMB was defined as total number of somatic non-synonymous mutations in coding region. The raw data that support the findings of this study are available from the corresponding author upon reasonable request. In addition, data from the Cancer Genome Atlas (TCGA, https://www.cbioportal.org/) was extracted in December 2018 [19,20]. Gene Oncology (GO) and pathway analysis on gene alterations from ctDNA were performed using DAVID (https://david.ncifcrf.gov/) with the parameters *p* value cutoff = 0.05, and drawn in R by using the package “ggplot”.

Demographic characteristics of patients were analyzed using the T test or Chi-Square (χ^2^) test. Two sided *p*-values were evaluated and *p* < 0.05 was regarded as significance with statistical meaning. All the statistical analyses were performed by SPSS software, version 20.0 (SPSS Inc^®^, United States).

## Results

### Patient Characteristics and Basic Features of Genomic Alterations

**FIGURE 1:**
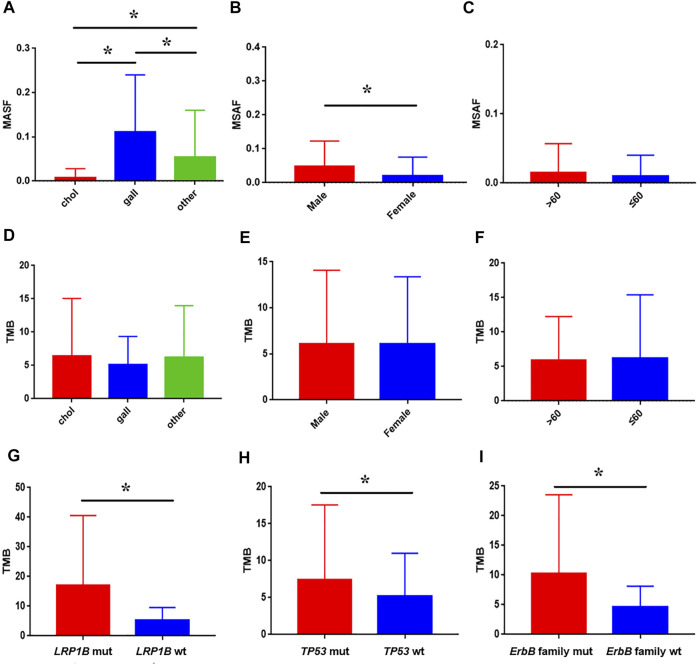
Association between baseline characteristics and ctDNA alteration in clinical samples. The impact of pathological subtype **(A)**, sex **(B)**, and age **(C)** on MSAF; the impact of pathological subtype **(D)**, sex **(E)**, and age **(F)** on TMB; comparison of TMB between patients with *LRP1B* **(G)**, *TP53* **(H)**, ErbB family **(I)** mutation and wild-type, respectively.

Hybrid capture-based genomic profiling were performed on ctDNA samples and tumor tissue DNA samples, respectively (Table 1). For patients who provided ctDNA samples, the median age was 61 years, ranging from 39 to 93 years old. Among them 66.2% were male. Cholangiocarcinoma was the most common pathologic subtype (72.1%), followed by gallbladder cancer (24.0%), and others (3.9%) such as ampullary carcinoma. ctDNA in the blood was detected in 94.8% of the cases as approximated using a maximum somatic allele frequency (MSAF) > 0. The median MSAF was 6.47% (range 0.1–34.8%) and the average number of GAs was 4. As shown in Figure 1, highest median MSAF was observed in patients with gallbladder cancer, followed by patients with other pathological types and those with cholangiocarcinoma (*p* < 0.05) (Figure 1A). Male patients showed significantly higher median MSAF compared to female patients (*p* = 0.0001) (Figure 1B) while age had no significant effect (Figure 1C). For patients who provided tumor tissue samples, the median age was 59 years, ranging from 19 to 83 years old. The gender composition is relatively close to balance, with a distribution of 56.1% male and 43.9% female. Similar pathological types were observed, and cholangiocarcinoma (67.3%) was the dominant one. For patients providing paired tumor tissue and blood samples, ctDNA in the blood was detected in 520 (95.4%) of them and the median MSAF of tissue DNA was a little higher than that of ctDNA. The average GAs was 5. No significant correlation was observed between diverse baseline characteristics and TMB, including pathological subtype, sex and age (Figures 1D–F).

**TABLE 1:** Characteristics of BTC patients who provided ctDNA or tissue samples.

Characteristic	ctDNA samples	Tissue samples
Cases	154	545
Median age, year (range)	61 (39–93)	59 (17–81)
Sex (male vs. female)	102 vs. 52	306 vs. 239
Subtype (cholangiocarcinoma vs. gallbladder carcinoma vs. other)	105 vs. 37 vs. 4	367 vs. 161 vs. 17
MSAF > 0, n (%)	146 (94.8%)	520 (95.4%)
Median MSAF	6.47% (0.1–34.8%)	19.9% (0.8–35.0%)
Average GA/case	4	5

MSAF, maximum somatic allele frequency; GA, genomic alteration.

### Genomic Alterations in Blood-Derived ctDNA

**FIGURE 2:**
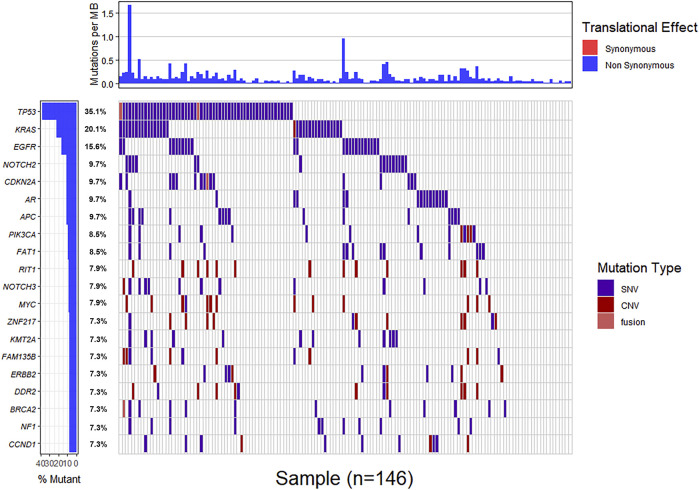
The mutation landscape of ctDNA samples. Mutations of genes in each sample were seen in the waterfall plot where various colors describing the specific forms of mutations were annotated.

**FIGURE 3:**
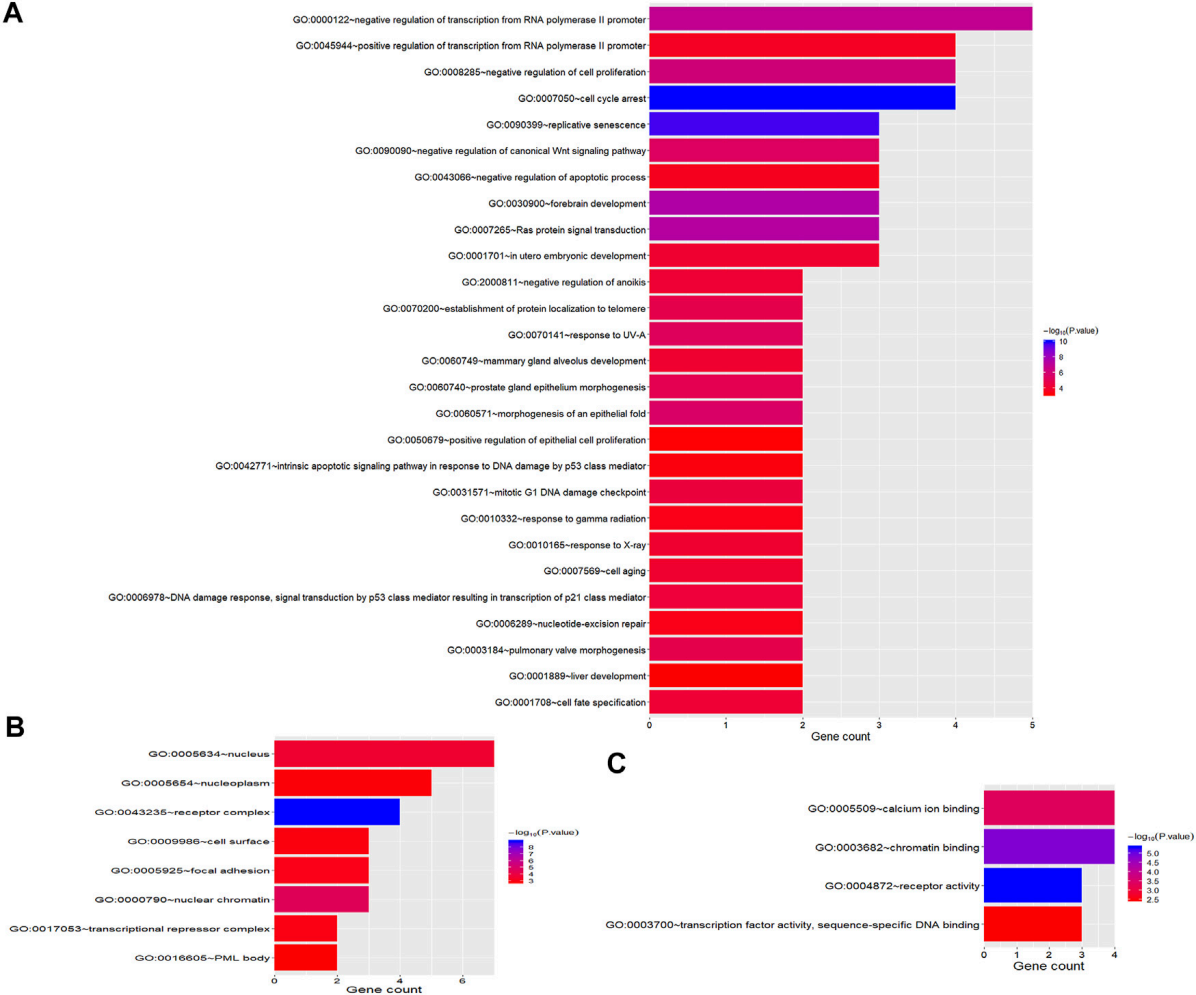
The mutation landscape of ctDNA in cholangiocarcinoma **(A)** and gallbladder **(B)** samples. Mutations of genes in each sample were seen in the waterfall plot where various colors describing the specific forms of mutations were annotated.

Genomic alterations in ctDNA samples were identified using unique barcoding markers (Figure 2). *TP53* (35.1%) and *KRAS* (20.1%) were found to be the most frequently altered genes. Using MutSigCV, other driver genes were also identified including *EGFR* (15.6%) and *CDKN2A* (9.7%). In 105 patients with cholangiocarcinoma, *TP53* was the most frequently altered gene in ctDNA, followed by *KRAS* and *EGFR* (Figure 3A). By contrast, 37 blood samples with gallbladder subtypes were significantly enriched for *TP53*, *CDKN2A*, and *EGFR* mutations (Figure 3B). Patients harboring *LRP1B*, *TP53*, and ErbB family mutations showed significantly higher tumor mutation burden (TMB, Figures 1G–I).

### GO Enrichment and Signaling Pathway Analysis of Genomic Alterations

**FIGURE 4:**
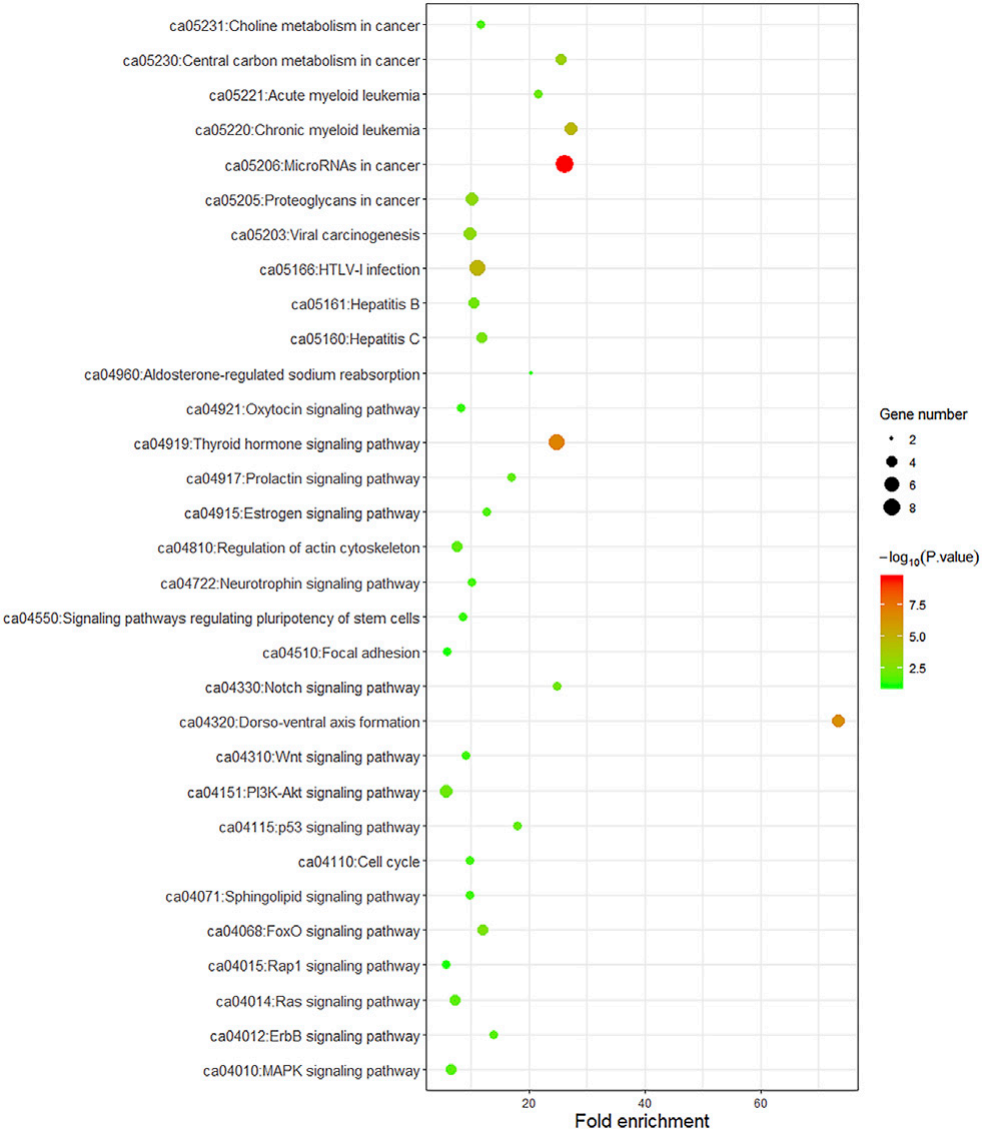
GO Enrichment Analysis of frequently mutated genes categorized by biological process **(A)**, cellular component **(B)** and molecular function **(C)**. The color represents the adjusted *p*-value.

**FIGURE 5:**
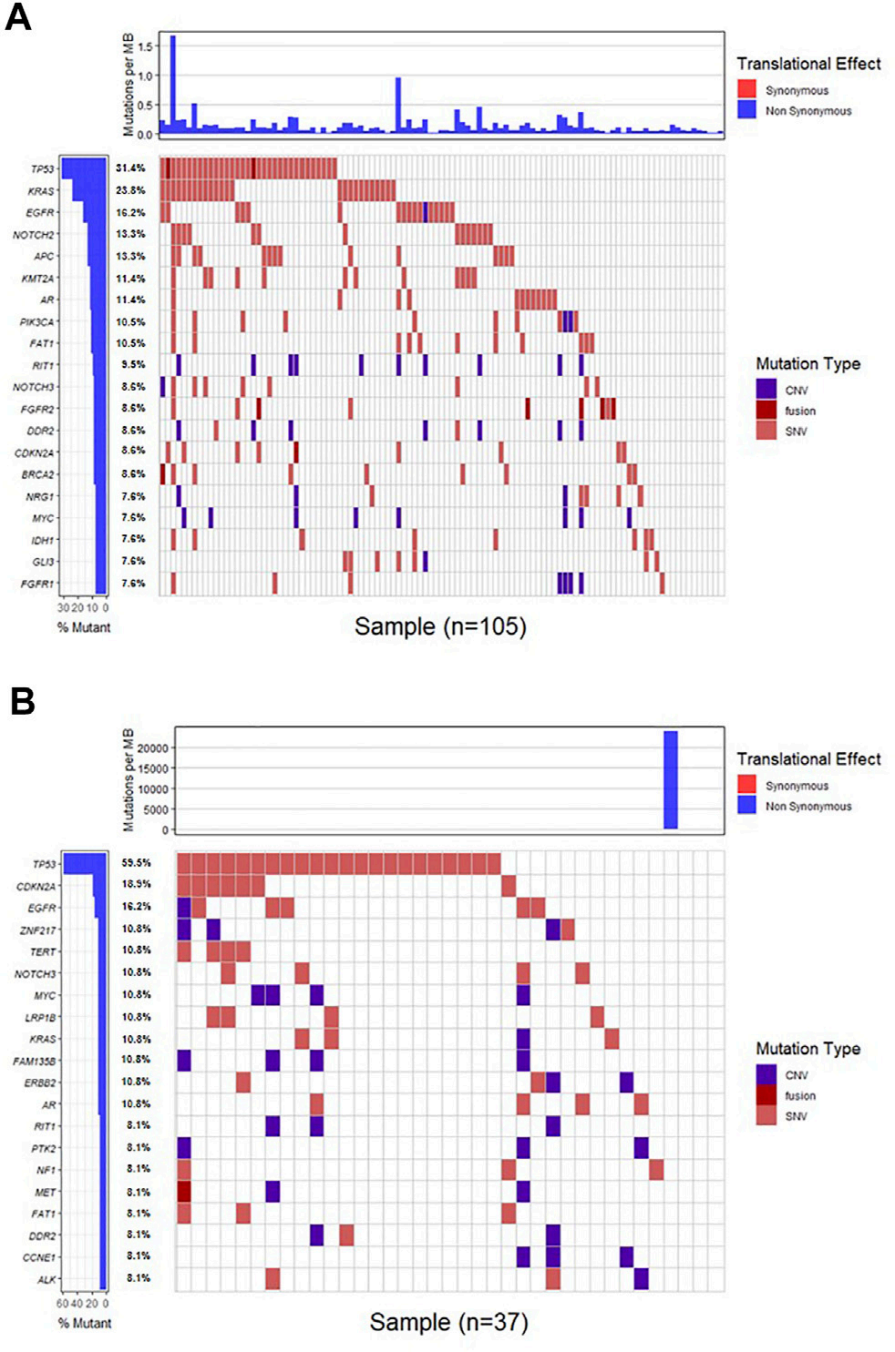
KEGG pathway enrichment dot plot of signaling pathways mapped by frequently mutated genes. The *y*-axis represents KEGG-enriched terms. The *x*-axis represents the fold of enrichment. The size of the dot represents the number of genes under a specific term. The color of the dots represents the adjusted *p*-value.

To better understand the biological function of these frequent alterations, gene ontology enrichment and signaling pathway analysis were performed. Figure 4 showed the significant enriched GO terms on three aspects, namely biological process (BP), cellular component (CC), and molecular function (MF). The top one enriched GO terms of BP was related to cell cycle and regulation, including regulation of transcription, regulation of cell proliferation, and cell cycle arrest. Most of the genes located in the nucleus, and the MF of calcium ion binding and chromatin binding enriched the most number of genes. As shown in Figure 5, a number of pathways that may be implicated in BTC were commonly mapped, including PI3K-Akt signaling pathway, p53 signaling pathway, ErbB signaling pathway, and Ras signaling pathway. These pathways has been recognized to be associated with tumorigenesis and reported to be frequently mutated in BTC [10,11].

### Comparison of Alterations in ctDNA Versus Tissue and TCGA Database

**FIGURE 6:**
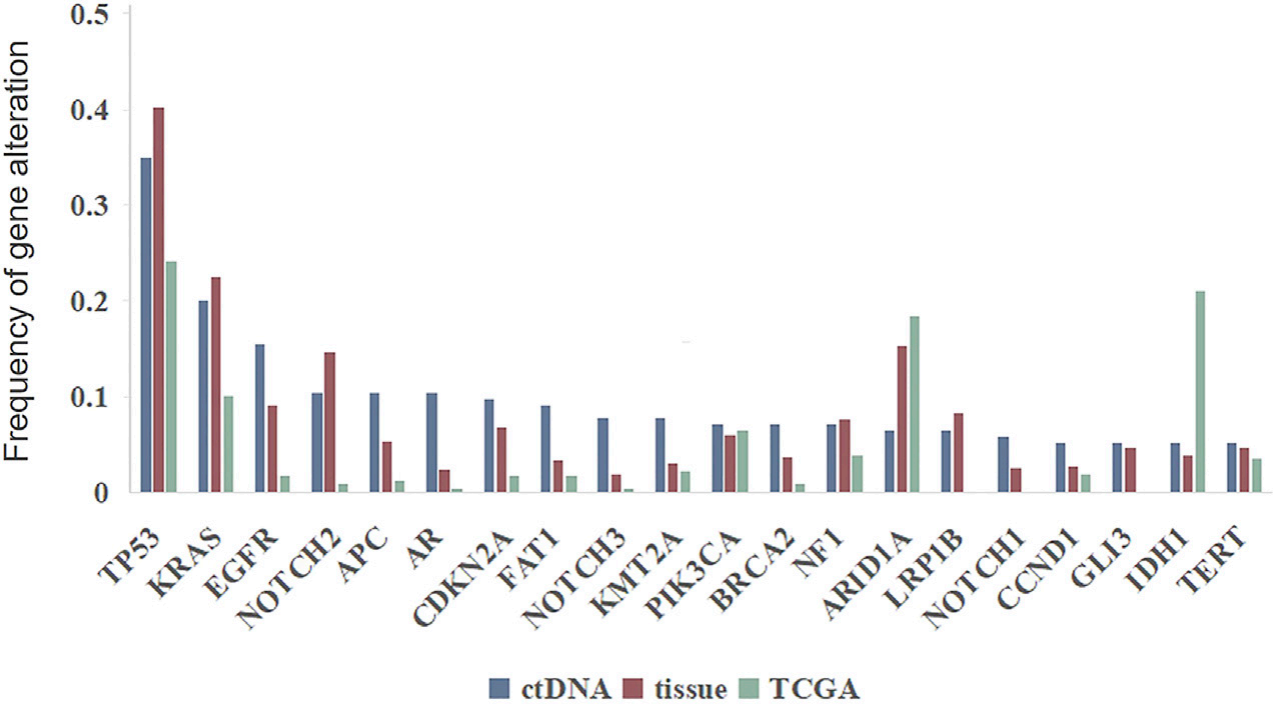
Genomic alterations in ctDNA versus in tumor DNA from clinical samples and TCGA database.

The frequencies of SNVs in commonly mutated genes in ctDNA samples were compared with the frequencies detected in tissue samples and TGCA database (Figure 6). *TP53* was the most commonly mutated gene in all the three data source (35.1 vs. 40.4% vs. 24.2%), followed by *KRAS* (20.1 vs. 22.6% vs. 10.1%). And for most genes the mutation frequencies in ctDNA were similar with those detected in tissue samples and were relative higher than in TCGA database, with the exception of *ARID1A* and *IDH1* which were most highly mutated in TCGA database.

## Discussion

Somatic mutations were analyzed in blood samples of patients with advanced BTC, and ctDNA somatic mutations could be detected in 94.8% of all the cases. This result is consistent with other publications. Oliver et al. [21] reported the fraction is 84.6% (22/26) in patients with pancreatobiliary carcinomas. In our study, *TP53* and *KRAS* were the most frequently mutated genes, followed by *EGFR*. A whole-exome and targeted gene sequencing result identified that genes with a significantly frequency of mutations included *TP53* (47.1%) and *KRAS* (7.8%); the ErbB signaling was the most extensively mutated pathway affecting 36.8% of the GBC patients [20]. *TP53* and *KRAS* were also identified as the significantly mutated genes in a cohort of ICC patients and Ras/PI3K signaling was one of the most affected pathways, followed by cell cycle signaling pathway [22]. These are basically consistent with our results on GO and pathway analysis.

*IDH1*, *FGFR2* and *BRAF* are targetable genetic alterations in BTC. In our cohort, the frequencies of *IDH1* mutations and *FGFR2* fusions detected by ctDNA profiling in cholangiocarcinoma were 7.4 and 4.8%, respectively. These were very close to the mutational frequencies in tissue-based testing, which were 6 and 2.7%, respectively. We did not see any case harboring *BRAF* V600E mutation, a rare occurrence restricted in intrahepatic cholangiocarcinoma [23]. These indicate that ctDNA analysis, an alternative for tissue analysis, might be helpful to guide clinical decision in advanced BTC.

We identified that there was no significant difference on TMB among diverse pathological subtypes, which is consistent with the previous publication [24]. We also found that patients with *LRP1B*, *TP53* or ErbB family member mutations had a significantly higher TMB than patients with wild-type genes respectively. *LRP1B* (low-density lipoprotein receptor-related protein 1B) gene mutations were frequently seen in multiple types of human cancer and had been recognized as driver mutations in liver cancer and pancreatic cancer [25–27]. Higher TMB was found in *LRP1B* mutated patients with melanoma and non-small cell lung cancer [28]. *TP53* is a key tumor suppressor gene. The encoded protein plays a key role in the regulation of cell cycle arrest, apoptosis, senescence, DNA repair and changes in metabolism. Mutations in this gene are associated with a variety of human cancers [29]. An integrated analysis on the genomic, transcriptomic, proteomic, and clinical data from cohorts of lung adenocarcinoma patients revealed that *TP53*-mutated tumors showed prominently increased mutation burden [30]. Although several researches have discussed the relationship between *LRP1B*/*TP53* gene mutation and TMB, no definite conclusions have been reached in BTC. The ErbB family of receptor tyrosine kinases comprises four members, ErbB-1/EGFR, ErbB-2/HER2, ErbB-3/HER3, ErbB-4/HER4. Mutation of these members occurred in nearly 15% of BTC patients and was associated with higher TMB [31]. This is consistent with our result.

The genomic landscape and molecular features of BTC have been reported in several papers [20,22,32]. However, almost all these researches use tumor tissues as the sequencing samples, and limited data on ctDNA profiling of BTC has been reported. On 2019 ASCO meeting, a research revealed the basic rudiment of the ctDNA genomic alteration landscape of BTC, and indicated that 55% of the patients harbored targetable genetic alterations [33]. Another blood-based genomic profiling also showed a subgroup of patients with BTC may benefit from targeted therapy and *TP53* and *KRAS* were the most frequently altered genes [34]. In these papers the sequencing panel was relative small, thus may limit our understanding of genomic features. Researchers from Germany analyzed the correlation between ctDNA alterations and disease progression in BTC using a 710 cancer-related-genes panel [35]. However, only eight patients were detected by this panel, making the admissibility of the result quite weak.

To our knowledge, this work firstly revealed the genomic landscape of ctDNA in BTC with a large sample size, and directly compared it with the genomic landscape of tumor tissue DNA. These results indicated that ctDNA could be used as a potential complementary tool for gene sequencing, aiding to screen patients who may benefit from targeted therapies.

One limitation of this paper is the lack of baseline clinical characteristics and therapeutic regimens. As the clinical features may affect the detection of ctDNA [36,37], further studies are needed to study the association between clinical information and molecular information.

## Data Availability

The datasets presented in this article are not readily available as the ethical approval did not cover public disclosure of the data. Requests to access the datasets should be directed to HS, tntboy1982@163.com.

## Abbreviations

BP, Biological process; BTC, Biliary tract cancer; CC, cfDNA Cellular component Cell-free DNA; CNV, Copy number variation; ctDNA, Circulating tumor DNA; GA, Genomic alteration; gDNA, Genomic DNA; GO, Gene oncology; ICI, Immune checkpoint inhibitor; LRP1B, Low-density lipoprotein receptor-related protein 1B; MF, Molecular function; MSAF, Maximum somatic allele frequency; SNV, Single nucleotide variant; TCGA, The Cancer Genome Atlas; TMB, Tumor mutation burden.
